# Associations between psychological distress and thyroid cancer, and the mediating role of lifestyle and metabolism: a cohort study from the UK Biobank

**DOI:** 10.3389/fonc.2026.1822262

**Published:** 2026-06-23

**Authors:** Yuan Liu, Meng-lin Fan, Qi-qi You, Jing-jing Zeng, Bo Chen, Wan Fu, Wen-xiang Yu, Shao-yong Xu

**Affiliations:** 1 Department of Oncology, Xiangyang Central Hospital, Affiliated Hospital of Hubei University of Arts and Science, Xiangyang, Hubei, China; 2 Department of Endocrinology, Xiangyang Central Hospital, Affiliated Hospital of Hubei University of Arts and Science, Xiangyang, Hubei, China; 3 Center for Clinical Evidence-Based and Translational Medicine, Xiangyang Central Hospital, Affiliated Hospital of Hubei University of Arts and Science, Xiangyang, Hubei, China; 4 Department of Neurology, Xiangyang Central Hospital, Affiliated Hospital of Hubei University of Arts and Science, Xiangyang, Hubei, China

**Keywords:** anxiety, depression, mediating factors, psychological distress, risk factors, thyroid cancer

## Abstract

**Background:**

Psychological distress increases cancer risk, but its relationship to thyroid cancer risk remains unclear. The aims of our study were to assess whether psychological distress increases the risk of thyroid cancer, and to further explore the mediating factors between psychological distress and thyroid cancer.

**Methods:**

A total of 420,187 participants aged 39–73 years between 2006 and 2010 in the UK biobank were included in the cohort study. Psychological distress was assessed using relevant hospitalization records, the four-item Patient Health Questionnaire and self-reported psychiatric symptoms. Thyroid cancer diagnosed date was from national cancer registries and hospital inpatient records in England, Wales, and Scotland. We used Cox proportional hazard models to calculate hazard ratios and the mediation R package to assess the mediating factors.

**Results:**

With a median follow-up of 12.59 years, the incidence of thyroid cancer in the participants with psychological distress was 137.16 per 100,000, compared with 94.31 per 100,000 in the participants with non-psychological distress. Psychological distress significantly increased the risk of thyroid cancer (HR, 1.33; 95% CI, 1.09–1.62; P = 0.005). Mediation analysis indicated that BMI, waist circumference, high-density lipoprotein, triglycerides, alcohol consumption partially mediated between psychological distress and thyroid cancer, in which waist circumference had the strongest mediating effect by a proportion of 11.80% (95% CI, 4.41–36.00%; P < 0.001).

**Conclusions:**

Psychological distress is associated with an increased risk of developing thyroid cancer, which is modestly mediated by obesity, dyslipidemia, and alcohol consumption. The hypothesis that managing weight, lipids, and alcohol intake might mitigate cancer risk in individuals with psychological distress requires direct testing in future intervention trials.

## Introduction

Thyroid cancer (TC) is the most common malignant tumor in the endocrine system. Based on the International Agency for Research on Cancer, the global number of new cases of TC in 2020 was 586,202 (1). From 1990 to 2019, the global age-standardized incidence of TC increased by 40.8% (2). Although overdiagnosis has been proposed to explain part of the observed increase in TC incidence worldwide (3, 4), it does not fully account for this trend (5). Therefore, the rising incidence of TC remains an important global public health issue.

Psychological distress is generally defined as a mental health disorder with symptoms of depression and anxiety (6), which leads to an increased risk of several chronic diseases, including cardiovascular disease, chronic obstructive pulmonary disease, and arthritis (7). The prevalence of psychological distress in the UK general population is as high as 15.1% (8). Notably, psychological distress can be measured and categorized according to the type of psychological problem and the severity of symptoms, regardless of diagnosis by a psychiatrist (9). This approach has gained increasing attention because of its wide applicability.

Biologically, psychological distress can lead to dysregulation of the hypothalamic–pituitary–adrenal (HPA) axis and sympathetic nervous system, and disruption of the immune and endocrine systems, which can adversely affect a variety of cancer defense processes (10, 11). There is growing evidence that psychological distress may increases cancer risk, such as lung, breast, colorectal, and prostate cancer (12, 13). However, the relationship between psychological distress and the risk of developing TC is unclear and related cohort studies are scarce. Two limited cohort studies found no significant relationship between psychological distress and the incidence of TC (14, 15). But, considering the fact that several previous cross-sectional studies suggested a positive association between psychological distress and TC (16, 17), and also the fact that fewer than 20 cases of thyroid cancer occurred in above two cohort studies, we hypothesized that the association has the possibility to be underestimated and large cohort studies are particular required.

Meanwhile, some studies have shown that people with chronically negative psychology are more likely to smoke or consume alcohol, be sedentary, be obese, and have insufficient sleep, and these lifestyle-related risk factors indirectly increase the likelihood of cancer (18, 19). We are also interested in factors that may mediate the relationship between psychological distress and TC. Given the aforementioned background, the aims of our study were to assess whether psychological distress increases the risk of TC and to further explore the mediating factors between psychological distress and TC based on the UK Biobank cohort.

## Methods

### Study design and population

The UK Biobank is a large and detailed prospective study that recruited more than 500,000 participants aged 39–73 years from 2006 to 2010. The cohort data were collected from questionnaires, physical measurements, sample analyses, accelerometry, multimodal imaging, genome-wide genotyping, and longitudinal follow-up for a wide range of health-related outcomes (20). The UK Biobank received ethical approval from the North-West Multi-center Research Ethics Committee. All participants provided written informed consent. The present study was performed under application number 92014.

**Figure 1:**
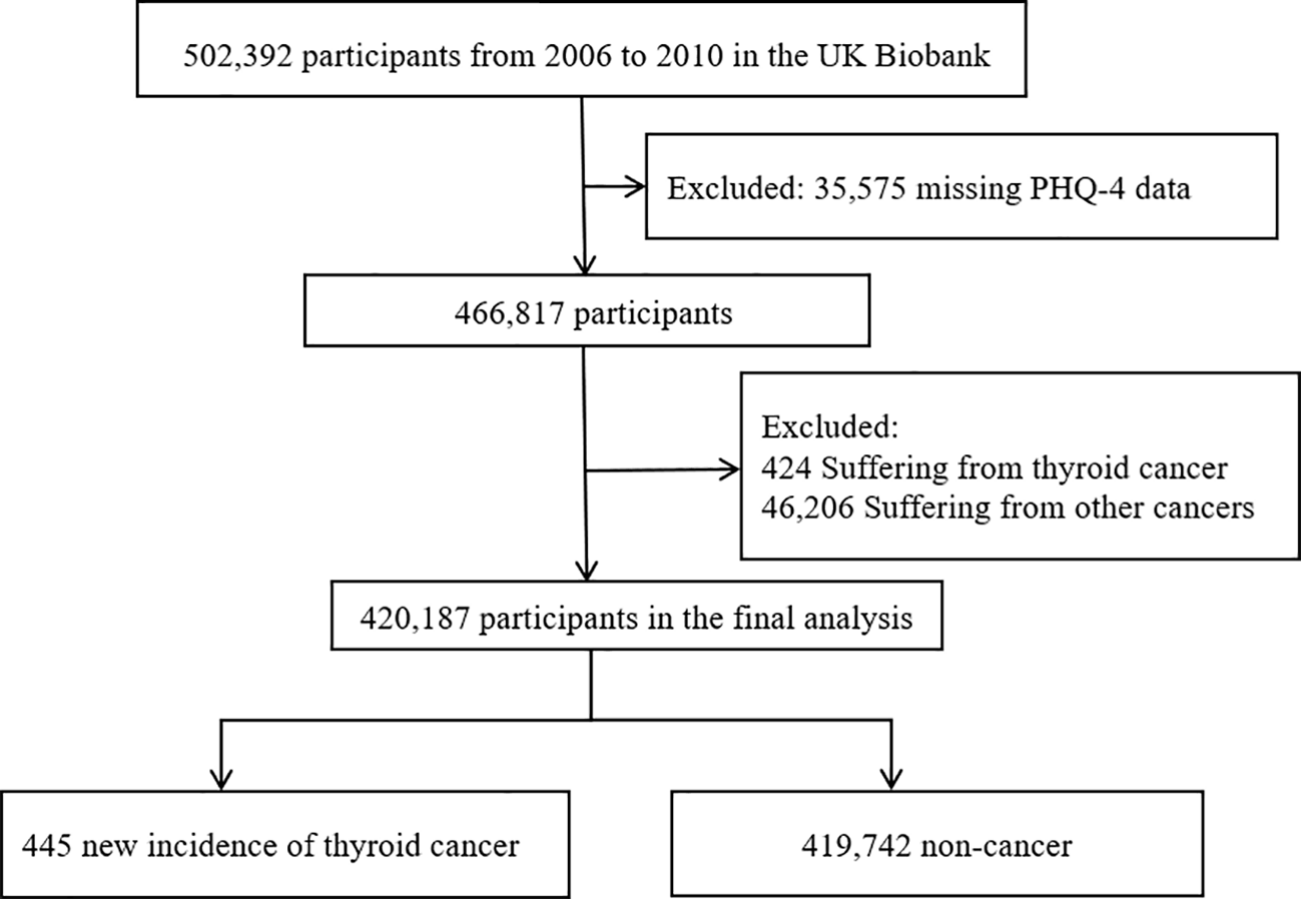
Study flowchart. PHQ-4, four-item Patient Health Questionnaire.

We excluded participants with missing data on the 4-item Patient Health Questionnaire (PHQ-4) (n = 35,575) and participants with TC at baseline (n = 424). To minimize the effect of reverse causality, we also excluded participants with other cancers at baseline (n = 46,206). A total of 420,187 participants ultimately entered the analysis (**Figure 1**), and followed up until September 30, 2021. This study is reported in accordance with the Strengthening the Reporting of Observational Studies in Epidemiology (STROBE) guidelines (**Supplementary Table 1**).

### Assessment of psychological distress

The study used multiple sources to determine psychological distress. Date and diagnosis were obtained through the primary care records, hospital admission records and self-reported medical conditions. Participants with diagnosis code for symptoms of depression and anxiety were defined as psychologically distressing [International Statistical Classification of Diseases and Related Health Problems, Tenth Revision (ICD-10): F32–F33 or anxiety (ICD-10: F40–F41)] (21). At baseline, symptoms of depression and anxiety were assessed using the PHQ-4 (22), which is a brief self-administered questionnaire consisting of a two-item depression scale (PHQ-2) and a two-item Generalized Anxiety Disorder (GAD-2). The PHQ−4 has been extensively validated as a reliable ultra−brief screening tool for depressive and anxiety symptoms, demonstrating good internal consistency (McDonald’s ω = 0.85) and strong criterion validity (23, 24). Participants were asked to rate their responses on a four-point Likert scale from 0 (not at all) to 3 (almost daily), their response to four items: 1. “frequency of depressed mood” (Data-Field: 2050), 2. “frequency of unenthusiasm/disinterest” (Data-Field: 2060), 3. “frequency of tenseness/restlessness” (Data-Field: 2070), and 4. “frequency of tiredness/lethargy” (Data-Field: 2080) (21). Total scores ranged from 0 to 12, with a total score ≥ 6 considered positive for depression/anxiety and defined as psychological distress. This cutoff corresponds to the 95th percentile in the general population as validated in large-scale normative studies (23, 24), and has been widely adopted in UK Biobank-based studies to define psychological distress (21). And, PHQ-2 ≥ 3 was considered positive for depression, and GAD-2 ≥ 3 was considered positive for anxiety. In addition, when asked “Have you ever seen a psychiatrist for nerves, anxiety, tension, or depression?” at baseline, participants who answered “yes” were recorded as having a prevalence of psychological distress.

### Diagnosis of TC

TC diagnosis and diagnosed date were identified by linking to national cancer registries and hospital inpatient records in England, Wales, and Scotland. TC was recorded in accordance with the ICD-10, code C73. Participants were accepted for follow-up until diagnosis of TC, death, loss to follow-up, or at the date of available data (September 30, 2021), whichever was earlier.

### Covariates

Sociodemographic information such as age, sex, ethnicity, and education were collected through questionnaires or verbal interviews at baseline. The Townsend deprivation index (TDI), a composite measure of poverty based on unemployment, non–car ownership, non–home ownership, and household overcrowding, was calculated based on national census data and assigned with the use of postal codes, with higher indices indicating higher levels of deprivation (25). Hyperthyroidism (E05), hypothyroidism (E03), and thyroid nodules (E04) were based on self-reported information and medical records.

Lifestyle information was based on self-reported information at baseline. In this study, seven modifiable behavioral factors were considered, comprising four traditional factors (smoking, alcohol consumption, physical activity, and diet) and three emerging factors (sleep duration, sedentary behavior, and social relationships) (26). Nonsmoking was categorized as healthy. Healthy drinking was defined as relatively regular moderate drinking (no more than one drink per day for women and two drinks per day for men; in the UK, one drink is standardized at 8 g of ethanol). Healthy physical activity was defined as at least 150 minutes of moderate activity or 75 minutes of vigorous activity or an equivalent combination per week. A healthy diet was defined as an adequate intake of at least half of the 10 food groups, which is recommended for a cardiometabolically healthy diet: increased consumption of fruits, vegetables, whole grains, fish, dairy products, and canola oil and decreased consumption of refined grains, processed/unprocessed meats, and sugar-sweetened beverages (27). Healthy sleep duration is 7–8 hours/day. Television viewing was used as a proxy for total recreational sedentary behavior, and a healthy level was defined as < 4 hours per day (28). Information on family size, frequency of visits from friends/family, and participation in leisure/social activities was used to assess the level of social connectedness, with a healthy level defined as not being socially isolated (29).

At the baseline assessment center visit, height, weight, waist circumference (WC), and blood pressure were measured by trained nurses. Body mass index (BMI) was classified into two categories according to World Health Organization standards: underweight/normal (< 25 kg/m^2^) and overweight/obese (≥ 25 kg/m^2^). Obesity for WC was considered at ≥ 94 and ≥ 80 cm for men and women, respectively (30). Systolic blood pressure ≥ 130 mmHg or diastolic blood pressure ≥ 85 mmHg was considered hypertensive. Blood collection and sampling procedures for this study have been previously described and validated (31).

### Statistical analysis

Categorical variables are reported as numbers and percentages, and continuous variables are reported as means (standard deviation). Comparisons between groups were estimated using Pearson’s χ2 test or the t test. The Kaplan–Meier curves were used to represent the cumulative incidence of TC by psychological distress and PHQ-4 score group.

The hazard ratio (HR) and 95% confidence interval (CI) for TC across psychological distress group were calculated using cox proportional hazards regression models. PHQ-4 scores were also used as a categorical variable (categorized into “0,” “1–6,” and “7–12” groups) and a continuous variable to assess the effect of the level of psychological distress on TC development. To evaluate the effect of symptoms of depression and anxiety on the development of TC, depression and anxiety scores on the PHQ-4 were categorized into three groups for analysis: “0,” “1–3,” and “4–6” groups.

Subgroups were analyzed according to sex, age, TDI, and BMI. To test the robustness of the results, some sensitivity analyses were performed. First, participants who developed TC within 2 years of follow-up were excluded to minimize reverse causality. Second, patients who developed other cancers during the follow-up period were excluded to minimize the role of other cancers. Third, patients with thyroid nodules, hyperthyroidism, and hypothyroidism were excluded.

The role of healthy lifestyle, obesity, metabolic abnormality, and C-reactive protein (CRP) in the association of psychological distress with TC was assessed through mediation analysis. Mediation analyses were conducted using the mediation R package to estimate the indirect, direct, and total effects of psychological distress on TC, in addition to the mediator share of healthy lifestyle, obesity, metabolic abnormality, and CRP (32).

All statistical analyses were performed using R (version 4.3.2) and SAS 9.4 (SAS Institute, Cary, NC, USA). P < 0.05 was considered statistically significant.

## Results

### Characteristics of study populations

Of the 420,187 participants, 196,794 (46.8%) were males, with a mean age of 56.2 ± 8.1 years. There were 113,736 (27.1%) participants who exhibited psychological distress at baseline. **Table 1** reports the baseline characteristics of participants by category of psychological distress. Participants experiencing psychological distress were more likely to be slightly younger, females, less educated, and more deprived. Meanwhile, psychologically distressed participants were more likely to have poor lifestyle behaviors (smoking, insufficient sleep, low levels of physical activity, sedentary behaviors, and poor social relationships), except for alcohol consumption.

**Table 1:** Baseline characteristics of UK Biobank participants according to psychological distress.

Characteristic	Total N = 420,187	Psychological distress		P value
		No	Yes	
		N = 306,451 (72.9)	N = 113,736 (27.1)	
Age	56.2 ± 8.1	56.5 ± 8.1	55.3 ± 8.0	<0.001
Sex, n (%)				<0.001
Male	196,794 (46.8)	151,015 (49.3)	45,779 (40.3)	
Female	223,393 (53.2)	155,436 (50.7)	67,957 (59.7)	
Townsend deprivation index	−1.3 ± 3.1	−1.6 ± 2.9	−0.6 ± 3.4	<0.001
Education, n (%)				<0.001
College or university	140,044 (33.3)	106,240 (34.7)	33,804 (29.7)	
A levels/AS levels or equivalent	47,432 (11.3)	34,990 (11.4)	12,442 (10.9)	
O levels/GCSEs, CSEs, or equivalent	110,311 (26.3)	80,456 (26.3)	29,855 (26.2)	
NVQ, HND, HNC, or equivalent	27,307 (6.5)	19,803 (6.5)	7,504 (6.6)	
Other	95,093 (22.6)	64,962 (21.2)	30,131 (26.5)	
Ethnicity, n (%)				<0.001
White	383,685 (91.3)	283,186 (92.4)	100,499 (88.4)	
Asian or Asian British	8,898 (2.1)	5,279 (1.7)	3,619 (3.2)	
Black or Black British	6,387 (1.5)	4,118 (1.3)	2,269 (2.0)	
Other ethnicities	21,217 (5.0)	13,868 (4.5)	7,349 (6.5)	
Smoking, n (%)				<0.001
Unhealthy	44,401 (10.6)	27,236 (8.9)	17,165 (15.2)	
Healthy	374,418 (89.4)	278,378 (91.1)	96,040 (84.8)	
Alcohol consumption, n (%)				<0.001
Unhealthy	121,585 (31.2)	91,258 (32.0)	30,327 (29.0)	
Healthy	267,951 (68.8)	193,686 (68.0)	74,265 (71.0)	
Diet, n (%)				0.664
Unhealthy	388,519 (92.5)	283,442 (92.5)	105,077 (92.5)	
Healthy	31,588 (7.5)	23,009 (7.5)	8,579 (7.5)	
Physical activity, n (%)				<0.001
Unhealthy	180,853 (45.6)	127,325 (43.7)	53,528 (51.0)	
Healthy	215,889 (54.4)	164,390 (56.4)	51,499 (49.0)	
Sleep, n (%)				<0.001
Unhealthy	133,862 (32.0)	87,003 (28.5)	46,859 (41.7)	
Healthy	284,156 (68.0)	218,683 (71.5)	65,473 (58.3)	
Sedentary behavior, n (%)				<0.001
Unhealthy	273,832 (65.2)	197,753 (64.6)	76,079 (67.0)	
Healthy	145,977 (34.8)	108,589 (35.4)	37,388 (33.0)	
Social connection, n (%)				<0.001
Unhealthy	37,964 (9.0)	23,010 (7.5)	14,954 (13.2)	
Healthy	382,144 (91.0)	283,441 (92.5)	98,703 (86.8)	
BMI	27.4 ± 4.8	27.2 ± 4.5	28.1 ± 5.4	<0.001
WC	90.4 ± 13.5	90.0 ± 13.1	91.4 ± 14.4	<0.001
DBP	82.2 ± 10.2	82.4 ± 10.1	81.9 ± 10.3	<0.001
SBP	137.6 ± 18.6	138.4 ± 18.6	135.5 ± 18.4	<0.001
FBG	5.1 ± 1.2	5.1 ± 1.2	5.2 ± 1.4	<0.001
HDL	1.4 ± 0.4	1.5 ± 0.4	1.4 ± 0.4	<0.001
TG	1.7 ± 1.0	1.7 ± 1.0	1.8 ± 1.1	<0.001
CRP	2.55 ± 4.26	2.40 ± 4.07	2.95 ± 4.73	<0.001
Hyperthyroid, n (%)				<0.001
No	419,318 (99.8)	305,934 (99.8)	113,384 (99.7)	
Yes	869 (0.2)	517 (0.2)	352 (0.3)	
Hypothyroid, n (%)				<0.001
No	415,074 (98.8)	303,589 (99.1)	111,485 (98.0)	
Yes	5,113 (1.2)	2,862 (0.9)	2,251 (2.0)	
Thyroid nodules, n (%)				<0.001
No	419,338 (99.8)	305,912 (99.8)	113,426 (99.7)	
Yes	849 (0.2)	539 (0.2)	310 (0.3)	

A/AS, advanced; O, ordinary; GCEs, General certificate of secondary educations; CSEs, Certificate of Secondary Educations; NVQ, National Vocational Qualification; HND, Higher National Diploma; HNC, Higher National Certificate; BMI, body mass index; WC, waist circumference; DBP, diastolic blood pressure; SBP, systolic blood pressure; FBG, fasting blood glucose; HDL, high-density lipoprotein; TG, triglycerides; and CRP, C-reactive protein.

### Association between psychological distress and the risk of TC

**Figure 2:**
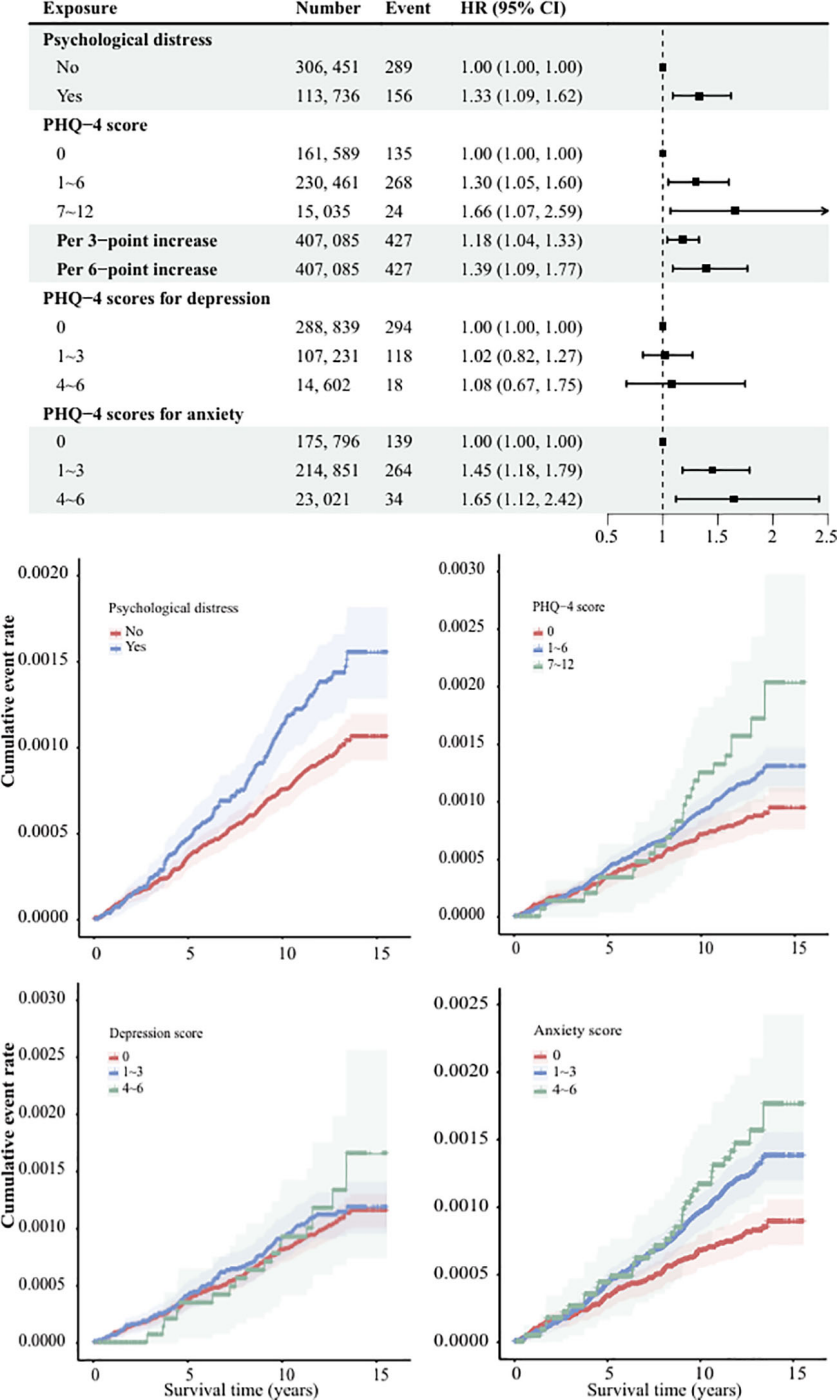
Association between psychological distress and the risk of thyroid cancer. The model adjusted for age, sex, ethnicity, education, Townsend deprivation index, and hyperthyroid, hypothyroid, and thyroid nodules.

During a median follow-up of 12.59 years (interquartile range 11.86–13.30 years), the incidence of TC in the participants with psychological distress was 137.16 per 100,000, compared with 94.31 per 100,000 in the participants with non-psychological distress. As presented in **Figure 2**, psychological distress increased the risk of TC, and this relationship remained significant in the fully adjusted model (HR, 1.33; 95% CI, 1.09–1.62; P = 0.005). Analysis of PHQ-4 scores as a continuous variable revealed that higher psychological distress scores were associated with a higher risk of TC in a dose–response relationship (HR_Per 6-point_ increase, 1.39; 95% CI, 1.09–1.77; P = 0.008). Participants in both the high scores group (HR, 1.65; 95% CI, 1.12-2.42; P = 0.011) and low scores group (HR, 1.45; 95% CI, 1.18-1.79; P < 0.001) of anxiety symptom had a significantly high risk of developing TC. In contrast, there was no significantly increased risk of TC in the high scores group (HR, 1.08; 95% CI,0.67-1.75; P = 0.756) and low scores group (HR, 1.02; 95% CI, 0.82-1.27; P = 0.847) of depressive symptoms.

**Figure 3:**
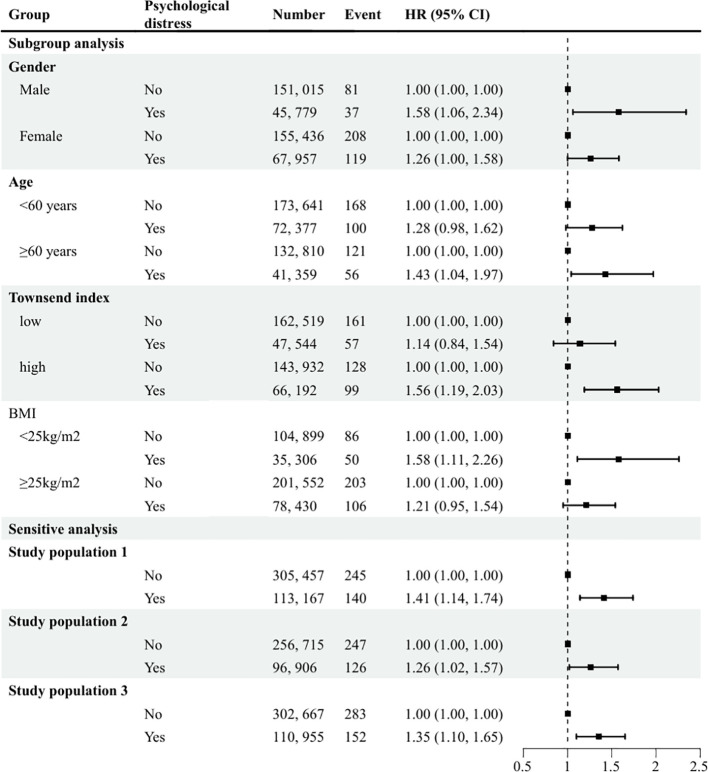
Subgroup analysis and sensitivity analysis. The model adjusted for age, sex, ethnicity, education, Townsend deprivation index, and hyperthyroid, hypothyroid, and thyroid nodules.

**Figure 3** presents the relationship between psychological distress and TC in stratified analyses based on age, sex, TDI, and BMI. We observed that psychological distress was positively associated with the risk of TC in both male (HR, 1.58; 95% CI, 1.06–2.34; P = 0.024) and female (HR, 1.26; 95% CI, 1.00–1.58; P = 0.049). Meanwhile, in the group of participants aged ≥ 60 years (HR, 1.43; 95% CI, 1.04–1.97; P = 0.030) with higher TDI (HR, 1.56; 95% CI, 1.19–2.03; P = 0.001) and with BMI < 25 kg/m^2^ (HR, 1.58; 95% CI, 1.11–2.26; P = 0.011), psychological distress was positively associated with the risk of TC. In sensitivity analyses, psychological distress was still associated with the risk of TC when excluding those who had TC within 2 years, those who had other cancers during the follow-up period, and those who had hyperthyroidism, hypothyroidism, or thyroid nodules at baseline (**Supplementary Table 2**).

### Mediation analysis

**Figure 4:**
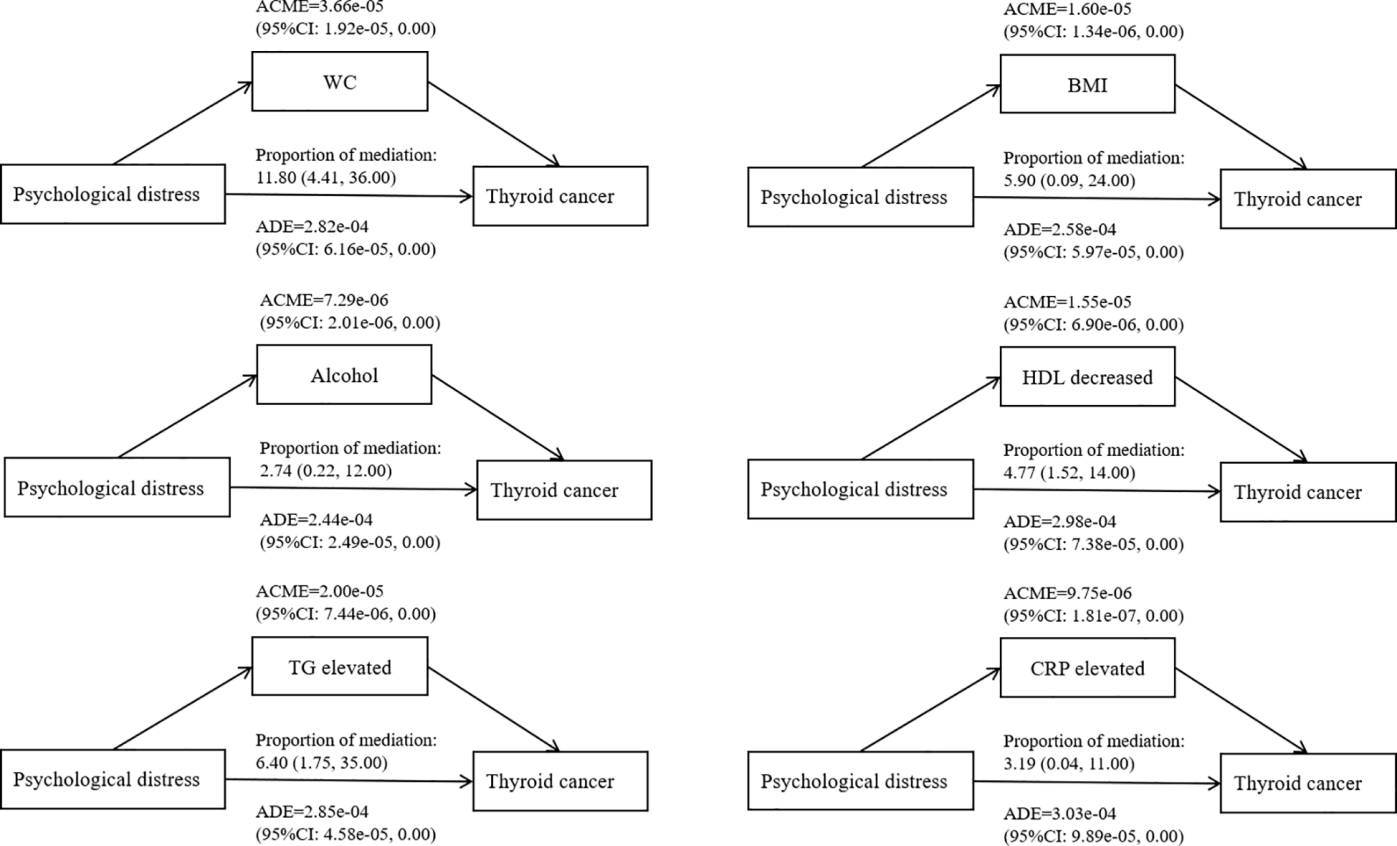
Mediation effects in the association between psychological distress and thyroid cancer. WC, waist circumference; BMI, body mass index; HDL, high-density lipoprotein; TG, triglycerides; CRP, C-reactive protein.

The results of the mediation analysis are presented in **Figure 4** and **Supplementary Tables 3–5**. Mediation analysis found that obesity, alcohol consumption, high-density lipoprotein (HDL), triglycerides (TG) and CRP partially mediated the association between psychological distress and TC. Psychological distress was significantly associated with obesity, particularly central obesity as measured by WC (Odd Ratio 9.71; 95% CI, 8.91–10.53; P < 0.001). Obesity was associated with the development of TC, with a similar risk for BMI (HR, 1.03; 95% CI, 1.01–1.05; P < 0.001) and WC (HR, 1.01; 95% CI, 1.01–1.02; P < 0.001). Mediation analysis revealed that WC had the strongest mediating effect by a proportion of 11.80% (95% CI, 4.41–36.00%; P < 0.001). In terms of lifestyle behaviors, only alcohol consumption played a weak mediating role at 2.74% (95% CI, 0.22–12.00%; P < 0.05) between psychological distress and TC, and the other factors did not influence the occurrence of TC. Dyslipidemia, which included decreased HDL or elevated TG, also exhibited some degree of mediation between psychological distress and TC.

## Discussion

To our knowledge, this is the first population-based cohort study with a large sample size on the relationship between psychological distress and TC. We observed that psychologically distressed participants had a 33% increased risk of TC, mainly because of the role played by symptoms of anxiety. Further mediation analysis revealed that obesity and dyslipidemia partially mediated the association between psychological distress and the development of TC. However, lifestyle behaviors did not mediate between the two, except for alcohol consumption.

Our study showed that participants with psychological distress had a significantly higher risk of TC, with inconsistent result from previous studies. However, one of the studies was based on a hospital-based population, with patients with a physician’s definitive diagnosis of anxiety or depression entered into the respective cohorts, with 11 cases of TC in the depression cohort and 9 cases of TC in the anxiety cohort (14). Another study was population-based with a median follow-up of 4.34 years and 18 cases of TC in the anxiety cohort (15). The reason for the inconsistency between the results of these two studies and ours may be related to differences in the study populations, sample sizes, follow-up duration, and definitions of psychological distress. In addition, previous studies on stress, temper tantrums and the risk of TC have found a direct correlation between them (33, 34), somewhat consistent with our findings. A Taiwanese population-based cohort study showed that physicians have a significantly higher risk of TC than the general population (35). Meanwhile, a correlation was found between the high prevalence of psychiatric disorders among Taiwanese physicians, particularly insomnia and anxiety (36), which indicated an association between anxiety and the risk of TC.

In addition, we found that anxiety exhibited a significantly higher risk of TC incidence than depression, which was consistent with previous studies on psychological distress and lung cancer risk (19, 37). Several pathways may explain this differential association. First, anxiety and depression differ in their neuroendocrine profiles. Anxiety is characterized by sustained HPA axis activation and sympathetic nervous system hyperarousal, whereas depression often involves HPA axis hypoactivation or blunted cortisol responses in chronic stages. Critically, a systematic review found that anxiety disorders are more consistently associated with dysregulation of the hypothalamic-pituitary-thyroid (HPT) axis, including elevated TSH and altered T4/T3 ratios, which could chronically stimulate thyroid follicular cells and increase proliferative risk (38). Second, emerging evidence suggests that chronic stress triggers gut dysbiosis, which promotes tumor progression through immune suppression and chronic inflammation (39). A large cross−sectional study confirmed that anxiety states significantly correlate with increased constipation risk, a proxy for altered gut transit and microbial composition (40). Anxiety may thus induce more pronounced changes in gut microbial composition than depression. Third, there are shared genetic substrates. The GABRB2 gene, associated with a variety of psychiatric disorders (41), is also hypomethylated and overexpressed in TC tissues (42, 43). Other genes such as ATF3 and the PI3K/AKT signaling pathway may also link psychiatric disorders to thyroid carcinogenesis (44). Given that in our study anxiety was significantly associated with thyroid cancer risk while depression was not, the effect may be driven primarily by anxiety−specific pathways rather than general distress. Future studies integrating multi−omics data are warranted to further disentangle these distinct mechanisms.

Our study further performed a series of mediation analyses for possible mediators, found that obesity and dyslipidemia were partially mediated the relationship between psychological distress and TC. A meta-study found that depression and anxiety were independently and positively associated with obesity indicators (45). HPA axis dysregulation, inflammatory factors, adipocytokines and adipokines, genetic correlations and high-fat diets may mediate the link between psychological distress and obesity (46–48). Previous studies have also shown that overweight and obesity were significantly associated with an elevated risk of TC (49, 50). Some studies have even shown that metabolic abnormalities increased the risk of TC development (51, 52). Its biological mechanisms are related to insulin resistance, thyroid stimulating hormone, adipocytokines and adipokines, and inflammatory factors (53). These mechanistic intersections may also laterally explain the partial mediating role of decreased HDL and elevated TG and CRP between psychological distress and the risk of TC. However, this study did not find an association between lifestyle behaviors and the risk of TC, thus, they could not serve as a mediator, except for alcohol consumption. This was inconsistent with the findings of a study that also used the UK Biobank to explore the correlation between lifestyle behaviors and TC, which assessed lifestyle behaviors in five areas: smoking, drinking, diet, exercise, and weight (54). However, the study excluded all participants with missing lifestyle variables, had a final enrollment of 264,956, and lacked adjustment for thyroid-related disorders in Model 2, which may have led to selection bias and confounding bias, increasing the strength of the association between lifestyle and TC.

The strengths of this study are population-based, long follow-up, a comprehensive definition of psychological distress, and adequate mediation analysis to find mediating factors between psychological distress and TC development. Our study also has limitations. First, the UK Biobank is predominantly of European populations and is not fully representative of the general population for a well-recognized “healthy volunteer” selection bias. Participants tend to be healthier, older, more likely to be female, and from less socioeconomically deprived areas (55). While internal validity of exposure−outcome associations is often robust to such bias, this selection bias would likely underestimate the true association between psychological distress and TC in the general population. Nevertheless, our findings should be cautiously extrapolated to other ethnic groups and populations because of genetic, environmental, and cultural differences. Second, although we adjusted for as many potential confounders as possible, data on iodine intake, radiation exposure, and family history were not available in the UK Biobank, and we therefore could not adjust for these established risk factors. Additionally, other unmeasured confounders may exist. Third, detailed information on histological subtypes was not available for the TC cases in our dataset. Therefore, we could not explore whether the association between psychological distress and TC risk differs by subtype, which is an important limitation for understanding potential disease heterogeneity. Fourth, we could not directly adjust for healthcare utilization, which may introduce detection bias, as individuals with psychological distress might have more frequent medical contacts leading to incidental thyroid cancer detection. Fifth, mediators were measured only at baseline, which may not reflect longitudinal changes. This limitation likely yields conservative effect estimates.

## Conclusions

Our study indicates that psychological distress is associated with an increased risk of developing thyroid cancer, and this association is partially mediated by obesity, dyslipidemia, and alcohol consumption. These findings may help raise public awareness of the potential link between mental health and physical outcomes, and support routine surveillance for thyroid cancer among individuals with psychological distress. Given the observational nature of our study, causation cannot be inferred. Therefore, the hypothesis that managing weight, lipids, and alcohol intake might mitigate cancer risk in distressed individuals requires direct testing in future intervention trials. Our results support the rationale for such investigations.

## Data Availability

Publicly available datasets were analyzed in this study. This data can be found here: Data are available in a public, open access repository. This research has been conducted using the UK Biobank Resource under Application Number 92014. The UK Biobank data are available on application to the UK Biobank (www.ukbiobank.ac.uk/) with access fees.
